# Association of the *GLB1* rs4678680 genetic variant with risk of HBV-related hepatocellular carcinoma

**DOI:** 10.18632/oncotarget.10963

**Published:** 2016-07-30

**Authors:** Wen-Tao Wang, Ziqiang Li, Meng Shi, Hui Zhu, Xiangyu Xiong, Jinhua Shang, Jibing Liu, Mujian Teng, Ming Yang

**Affiliations:** ^1^ Department of Hepatobiliary Surgery, Qianfoshan Hospital, Shandong University, Jinan, Shandong Province, China; ^2^ Shandong Provincial Key Laboratory of Radiation Oncology, Cancer Research Center, Shandong Cancer Hospital Affiliated to Shandong University, Shandong Academy of Medical Sciences, Jinan, Shandong Province, China; ^3^ College of Life Science and Technology, Beijing University of Chemical Technology, Beijing, China; ^4^ Department of Radiation Oncology, Shandong Cancer Hospital Affiliated to Shandong University, Shandong Academy of Medical Sciences, Jinan, Shandong Province, China; ^5^ Department of Intervention Surgery, Shandong Cancer Hospital Affiliated to Shandong University, Shandong Academy of Medical Sciences, Jinan, Shandong Province, China

**Keywords:** GLB1, genetic polymorphism, HBV, HCC, susceptibility

## Abstract

Accumulated evidences demonstrated that GLB1 is involved in cell senescence and cancer development. The *GLB1* rs4678680 single nucleotide polymorphism (SNP) has been identified as a hepatocellular carcinoma (HCC) susceptibility polymorphism by a genome-wide association study in Korean population previously. However, little or nothing was known about its involvement and functional significance in hepatitis B viruses (HBV)-related HCC in Chinese. Therefore, we investigated the association between the *GLB1* rs4678680 SNP and HBV-related HCC risk as well as its biological function *in vivo*. Genotypes were determined in two independent case-control sets from two medical centers of China. Odds ratios (ORs) and 95% confidence intervals (CIs) were estimated by logistic regression. The potential regulation role the rs4678680 genetic variant on *GLB1* expression was examined with HCC and normal liver tissues. We found that The rs4678680 G allele was showed to be risk allele; individuals with the TG genotype had an OR of 1.51 (95% CI = 1.10–2.07, *P* = 0.010, Shandong set) or 1.49 (95% CI = 1.11–1.99, *P* = 0.008, Jiangsu set) for developing HBV-related HCC, respectively, compared with individuals with the TT genotype. This association was more pronounced in males, individuals aged older than 57 years and drinkers (all *P* < 0.05). In the genotype-phenotype correlation analyses of fifty-six human liver tissue samples, rs4678680 TG or GG was associated with a statistically significant increase of *GLB1* mRNA expression (*P* < 0.05). Our data indicated that the *GLB1* rs4678680 SNP contributes to susceptibility to develop HBV-related HCC, highlighting the involvement of *GLB1* and cell senescence in etiology of HCC.

## INTRODUCTION

Although ranking the fifth most common cancer, hepatocellular carcinoma (HCC) is the third leading cause of cancer-related mortality worldwide [1]. There is a high incidence of HCC in China and other Asia-Pacific region [1]. Notably, China alone accounts for about 50% of all HCC cases in the world [1, 2]. There were several epidemiological features, such as marked variations between geographical regions, racial and ethnic groups, and sex. It has been revealed that men have a higher prevalence of HCC than women, i.e. the male:female ratio of HCC patients is ~2.65:1 in high-risk Chinese populations [2, 3]. Chronic infections with the hepatitis B or C viruses (HBV or HCV), exposure to dietary aflatoxin B as well as excessive alcohol drinking have been identified as major environmental risk factors of HCC [2, 3]. Since about 75% cases of HCC are associated with HBV infection in China [4], HBV infection is considered as one major risk factor for HCC. However, only 15% chronic HBV carriers suffer from HCC in their lifetime [2, 3], indicating that host genetic factors may also be involved in development of HBV-related HCC. This notion has been proved by several candidate gene and genome-wide association study (GWAS) on HBV-related HCC in eastern Asians [5–10].

To identify genetic factors associated with HCC, Clifford et al. conducted a comprehensive, genome-wide variation analysis (both genetic polymorphisms and copy number variations) in a population of unrelated Asian individuals [11]. After genotyping 386 Korean HCC cases and 587 Korean controls with the Affymetrix SNP6.0 assay, they found that the 3p21.33 *GLB1* rs4678680 G > T polymorphism is most significantly associated with HCC risk (odds ratio [OR] = 2.27, 95% confidence intervals [CI] = 1.68–3.08, *P* = 6 × 10^−7^). *GLB1* encodes a member of the glycosyl hydrolase 35 family of proteins, which catalyzes the hydrolysis of a terminal betalinked galactose residue from ganglioside substrates and other glycoconjugates. Accumulated evidences demonstrated that GLB1 is involved in cell senescence [12, 13] and cancer development [13–16]. Senescence phenotype could be induced by increased SA-b-galactosidase staining and elevated *GLB1* mRNA expression, indicating the important role of GLB1 in cell senescence. During the long latency of tumor development, oncogene-induced senescence has emerged as a barrier to tumorigenesis including HCC. Chen et al. conducted a replication case-control study on the association of 1p36.22, 2q32.2–q32.3, 3p21.33, 8p12, 14q32.11 and 21q21.3 with HCC among 507 Chinese HCC patients and 3014 Chinese controls. However, they did not included *GLB1* rs4678680 G > T single nucleotide polymorphism (SNP) in the study. As a result, it is still largely unclear if *GLB1* rs4678680 polymorphism plays a part in etiology of HCC in Chinese. Also, little or nothing has been known about functional significance of the *GLB1* rs4678680 SNP in HBV-related HCC. Therefore, we conducted two large independent case-control studies to investigate the association between the *GLB1* rs4678680 genetic polymorphism and risk for developing HBV-related HCC. To validate the biological function of the rs4678680 SNP *in vivo*, we examined the association between *GLB1* rs4678680 genotypes and its mRNA expression levels in normal liver tissues.

## RESULTS

Allele frequencies and genotype distributions of *GLB1* rs4678680 SNP in cases and controls from the Shandong and Jiangsu sets are showed in Table 1. The allele frequencies for rs4678680 G were 0.087 or 0.094 in cases and 0.059 or 0.058 in controls in Shandong or Jiangsu case-control set. All observed genotype frequencies in both cases and controls conform to Hardy-Weinberg equilibrium. Distributions of the rs4678680 genotypes were compared between HCC cases and controls. Frequencies of rs4678680 TT, TG and GG genotypes among HCC cases differed significantly from those among controls in either Shandong set (χ^2^ = 7.593, *P* = 0.022, *df* = 2) or Jiangsu set (χ^2^ = 16.14, *P* = 3.12 × 10^−4^, *df* = 2).

**Table 1:** Genotype frequencies of the *GLB1* rs4678680 G > T polymorphism among HCC cases and chronic HBV carriers and its association with HBV-related HCC risk

Studies	Genotypes	HCC cases No. (%)	Chronic HBV carriers No. (%)	OR^a^ (95% CI)	*P*-value^a^
		*n* = 1186	*n* = 508		
	TT	987 (83.2)	449 (88.4)	Reference	
Shandong set	TG	192 (16.2)	58 (11.4)	1.51 (1.10–2.07)	0.010
	GG	7 (0.6)	1 (0.2)	NC	
	TG + GG	199 (16.8)	59 (11.6)	1.53 (1.12–2.10)	0.007
		*n* = 620	*n* = 1200		
	TT	509 (82.1)	1062 (88.4)	Reference	
Jiangsu set	TG	105 (16.9)	136 (11.3)	1.49 (1.11–1.99)	0.008
	GG	6 (1.0)	2 (0.2)	NC	
	TG + GG	111 (17.9)	138 (11.5)	1.57 (1.18–2.09)	0.002
		*n* = 1806	*n* = 1708		
	TT	1496 (82.8)	1511 (88.5)	Reference	
Pooled	TG	297 (16.5)	194 (11.4)	1.56 (1.24–1.97)	1.76 × 10^−4^
	GG	13 (0.7)	3 (0.2)	NC	
	TG + GG	310 (17.2)	197 (11.6)	1.52 (1.19–1.94)	0.001

Note: HBV, hepatitis B virus; HCC, hepatocellular carcinoma; OR, odds ratio; CI, confidence interval; NC, not calculated.

a HCC case vs. chronic HBV carriers, data were calculated by logistic regression with adjustment for age, sex, smoking and drinking.

Unconditional logistic regression analysis was used to examine associations between the *GLB1* rs4678680 SNP and HBV-related HCC risk in Shandong and Jiangsu sets (Table 1). The rs4678680 G allele was showed to be risk allele; individuals with the TG genotype had an OR of 1.51 (95% CI = 1.10–2.07, *P* = 0.010, Shandong set) or 1.49 (95% CI = 1.11–1.99, *P* = 0.008, Jiangsu set) for developing HBV-related HCC, respectively, compared with individuals with the TT genotype (Table 1). It was also found that carriers of the rs4678680 TG or GG genotype showed significantly and consistently increased risk to develop HBV-related HCC compared with the TT carriers in both case-control sets (Shandong set: OR = 1.53, 95% CI = 1.12–2.10, *P* = 0.007; Jiangsu set: OR = 1.57, 95% CI = 1.18–2.09, *P* = 0.002). In the pooled analyses, we observed that the odds of having the rs4678680 TG genotype in cases was 1.56 (95% CI = 1.24–1.97, *P* = 1.76 × 10^−4^) compared with the TT genotype. Similarly, the rs4678680 TG or GG genotype carriers showed a 1.52-fold increased HCC risk compared with the TT genotype carriers (95% CI = 1.19–1.94, *P* = 0.001).

The risk of HBV-related HCC associated with the *GLB1* rs4678680 genotypes was further examined by stratifying for sex (Table 2). A significantly increased risk of HCC associated with the rs4678680 TG or GG genotype compared with the TT genotype was observed in males (Shandong set: OR =1.60, 95% CI = 1.15–2.23; *P* = 0.006; Jiangsu set: OR = 1.59, 95% CI = 1.17–2.16; *P* = 0.003). However, this genetic polymorphism was not significantly associated with HCC risk in females (all *P* > 0.05). In the stratification analyses with age, elevated risk of HCC associated with the *GLB1* rs4678680 TG or GG genotype was only observed among individuals aged older than 57 years (Shandong set: OR = 1.63, 95% CI = 1.01–2.62; *P* = 0.047), but not among individuals aged 57 years or younger (Shandong set: OR = 1.46, 95% CI = 0.96–2.21; *P* = 0.076) (Table 3). Similar results were observed among individuals aged older than 57 years in Jiangsu set (rs4678680 TG or GG genotype: OR = 2.10, 95% CI = 1.37–3.21; *P* = 0.001). Interestingly, the rs4678680 TG or GG genotype was also significantly associated with increased HCC risk in drinkers compared to the TT genotype in both sets (Shandong: OR = 1.90, 95% CI = 1.26–2.86, *P* = 0.002; Jiangsu: OR = 1.76, 95% CI = 1.21–2.57, *P* = 0.003) (Table 4). However, no such associations was observed among non-drinkers (all *P* > 0.05).

**Table 2:** Risk of HBV-related HCC associated with *GLB1* rs4678680 G > T genotypes by sex

Studies	Genotypes	Males				Females			
		Cases No. (%)	Controls No. (%)	OR^a^ (95% CI)	*P*-value^a^	Cases No. (%)	Controls No. (%)	OR^a^ (95% CI)	*P*-value^a^
		*n* = 1018	*n* = 425			*n* = 168	*n* = 83		
	TT	848 (83.3)	373 (87.8)	Reference		139 (82.7)	76 (91.6)	Reference	
Shandong set	TG	164 (16.1)	51 (12.0)	**1.58 (1.13–2.21)**	**0.008**	28 (16.7)	7 (8.4)	1.39 (0.54–3.60)	0.500
	GG	6 (0.6)	1 (0.2)	NC		1 (0.6)	0 (0)	NC	
	TG + GG	170 (16.7)	52 (12.2)	**1.60 (1.15–2.23)**	0.006	29 (17.3)	7 (8.4)	1.47 (0.57–3.77)	0.428
		*n* = 531	*n* = 998			*n* = 89	*n* = 202		
	TT	437 (82.3)	884 (88.6)	Reference		72 (80.9)	178 (88.1)	Reference	
Jiangsu set	TG	88 (16.6)	112 (11.2)	**1.50 (1.11–2.05)**	**0.011**	17 (19.1)	24 (11.9)	1.36 (0.38–4.83)	0.639
	GG	6 (1.1)	2 (0.2)	NC		0 (0)	0 (0)	NC	
	TG + GG	94 (17.7)	114 (11.4)	**1.59 (1.17–2.16)**	**0.003**	17 (19.1)	24 (11.9)	1.36 (0.38–4.83)	0.639

Note: HBV, hepatitis B virus; HCC, hepatocellular carcinoma; OR, odds ratio; CI, confidence interval; NC, not calculated.

a HCC case vs. chronic HBV carriers, data were calculated by logistic regression with adjustment for age, smoking and drinking.

**Table 3:** Risk of HBV-related HCC associated with *GLB1* rs4678680 G >T genotypes by age

Studies	Genotypes		Age	(≤ 57 years)			Age	(> 57 years)	
		Cases No. (%)	Controls No. (%)	OR^a^ (95% CI)	*P*-value^a^	Cases No. (%)	Controls No. (%)	OR^a^ (95% CI)	*P*-value^a^
		*n* = 627	*n* = 262				*n* = 559	*n* = 246	
	TT	516 (82.3)	228 (87.0)	Reference		471 (84.3)	221 (89.8)	Reference	
Shandong set	TG	108 (17.2)	33 (12.6)	1.46 (0.96–2.23)	0.077	84 (15.0)	25 (10.2)	1.56 (0.96–2.51)	0.072
	GG	3 (0.5)	1 (0.4)	NC		4 (0.7)	0 (0)	NC	
	TG + GG	111 (17.7)	34 (13.0)	1.46 (0.96–2.21)	0.076	88 (15.7)	25 (10.2)	**1.63 (1.01–2.62)**	**0.047**
		*n* = 315	*n* = 633			*n* = 305	*n* = 567		
	TT	265 (84.1)	552 (87.2)	Reference		244 (80.0)	510 (89.9)	Reference	
Jiangsu set	TG	44 (14.0)	79 (12.5)	1.06 (0.70–1.60)	0.791	61 (20.0)	57 (10.1)	**2.10 (1.37–3.21)**	**0.001**
	GG	6 (1.9)	2 (0.3)	NC		0 (0)	0 (0)	NC	
	TG + GG	50 (15.9)	81 (12.8)	1.19 (0.80–1.78)	0.388	61 (20.0)	57 (10.1)	**2.10 (1.37-3.21)**	**0.001**

Note: HBV, hepatitis B virus; HCC, hepatocellular carcinoma; OR, odds ratio; CI, confidence interval; NC, not calculated.

a HCC case vs. chronic HBV carriers, data were calculated by logistic regression with adjustment for sex, smoking and drinking.

**Table 4:** Risk of HBV-related HCC associated with *GLB1* rs4678680 G > T genotypes by alcohol drinking

Studies	Genotypes	Nondrinkers				Drinkers			
		Cases No. (%)	Controls No. (%)	OR^a^ (95% CI)	*P*-value^a^	Cases No. (%)	Controls No. (%)	OR^a^ (95% CI)	*P*-value^a^
		*n* = 410	*n* = 195			*n* = 776	*n* = 313		
	TT	349 (85.1)	168 (86.2)	Reference		638 (82.2)	281 (89.8)	Reference	
Shandong set	TG	59 (14.4)	26 (13.3)	1.16 (0.70–1.92)	0.578	133 (17.2)	32 (10.2)	**1.82 (1.21–2.75)**	**0.004**
	GG	2 (0.5)	1 (0.5)	NC		5 (0.6)	0 (0)	NC	
	TG+GG	61 (14.9)	27 (13.8)	1.14 (0.69–1.87)	0.620	138 (17.8)	32 (10.2)	**1.90 (1.26–2.86)**	**0.002**
		*n* = 156	*n* = 706			*n* = 464	*n* = 494		
	TT	133 (85.3)	629 (89.1)	Reference		376 (81.1)	433 (87.7)	Reference	
Jiangsu set	TG	20 (12.8)	76 (10.8)	1.25 (0.68–2.29)	0.468	85 (18.3)	60 (12.1)	**1.72 (1.17–2.51)**	**0.006**
	GG	3 (1.9)	1 (0.1)	NC		3 (0.6)	1 (0.2)	NC	
	TG + GG	23 (14.7)	77 (10.9)	1.42 (0.79–2.53)	0.240	88 (18.9)	61 (12.3)	**1.76 (1.21–2.57)**	**0.003**

Note: HBV, hepatitis B virus; HCC, hepatocellular carcinoma; OR, odds ratio; CI, confidence interval; NC, not calculated.

a HCC case vs. chronic HBV carriers, data were calculated by logistic regression with adjustment for age, sex and smoking.

**Figure 1:**
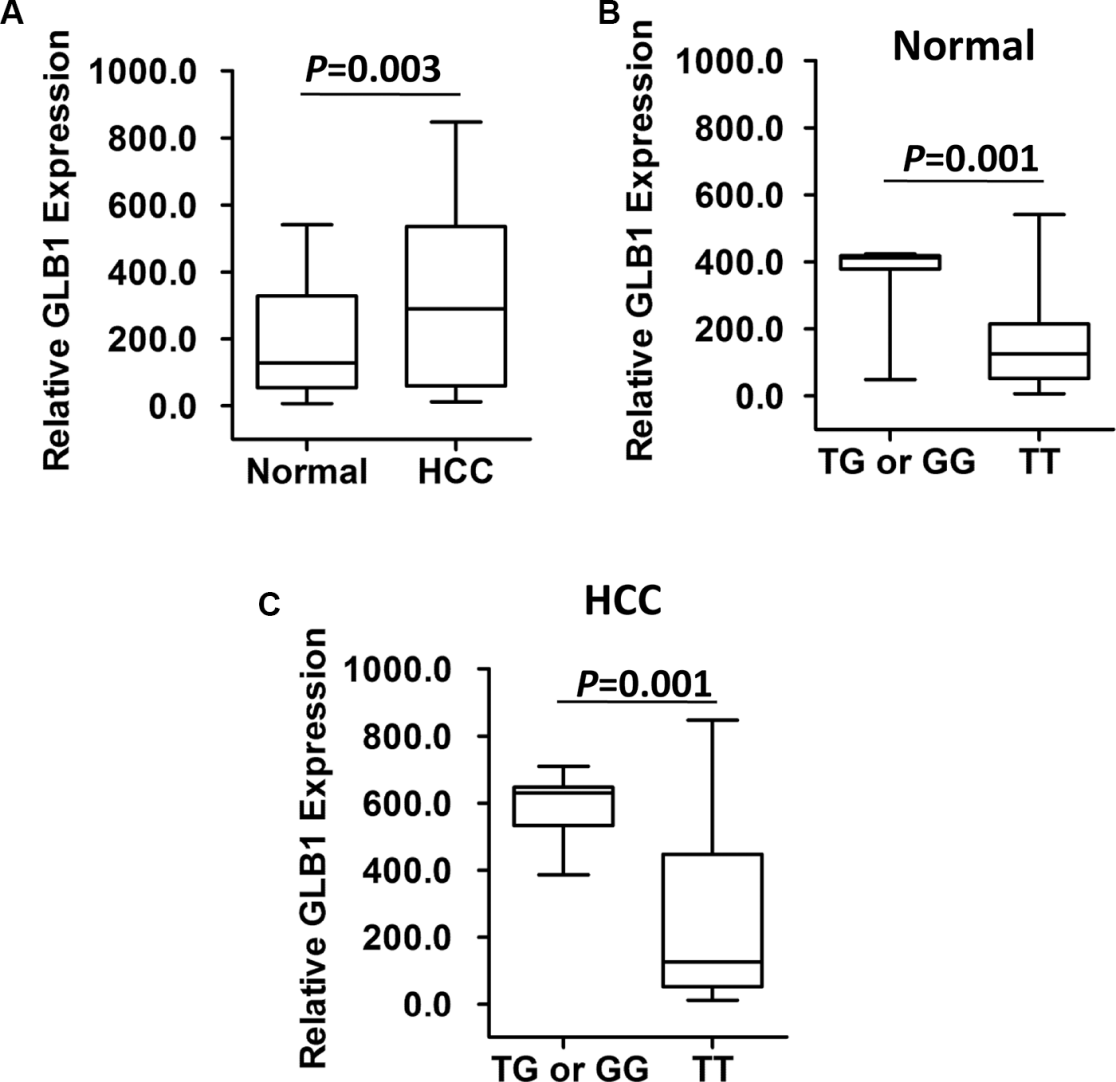
*GLB1* mRNA expression in fifty-six pairs of HCC-normal liver tissues (**A**) There were higher *GLB1* mRNA expression in HCC tissues than those in normal liver tissues. (**B**) *GLB1* mRNA expression in normal liver tissues grouped by *GLB1* rs4678680 genotypes. (**C**) *GLB1* mRNA expression in HCC tissues grouped by *GLB1* rs4678680 genotypes.

We next examined whether the HCC susceptibility SNP rs4678680 has an allele-specific impact on *GLB1* expression using HCC tissues since it locates in a 18 kb upstream region of *GLB1*. As shown in Figure 1, we found that there were significantly higher *GLB1* mRNA levels (mean ± SD) in HCC tissues compared to normal tissues (313.3 ± 33.93 vs. 189.9 ± 21.1; *P* = 0.003). Subjects with the rs4678680 TG or GG genotype had significantly higher *GLB1* mRNA levels than those with the TT genotypes in normal tissues (TG or GG: 367.5 ± 40.2 [*n* = 9], TT: 155.9 ± 20.6 [*n* = 47]; *P* = 0.001). Similar results were observed when the *GLB1* mRNA levels were compared between rs4678680 TG or GG and TT genotypes in HCC tissues (TG or GG: 590.7 ± 32.2 [*n* = 9], TT: 260.1 ± 35.0 [*n* = 47]; *P* = 0.001) (Figure 1).

## DISCUSSION

In the current study, we examined the association between the *GLB1* rs4678680 SNP and risk of developing HBV-related HCC in a case-control design. Although the genetic predisposition to HCC of the *GLB1* rs4678680 polymorphism was firstly identified in Korean populations via GWAS, this is still the first validation study with relative large sample size in different ethnic populations. We found significantly increased HCC risk among individuals with the *GLB1* rs4678680 TG or GG genotype compared with those with TT genotype in Chinese. In the genotype-phenotype correlation analyses of fifty-six human liver tissue samples, rs4678680 TG or GG was associated with a statistically significant increase of *GLB1* mRNA expression.

The *GLB1* gene provides instructions for producing an enzyme called β-galactosidase. This enzyme is located in lysosomes, which are compartments within cells that break down and recycle different types of molecules. Within lysosomes, GLB1 helps break down certain molecules, including substances called GM1 ganglioside and keratan sulfate. Caldwell et al. found that GLB1 activity is the only biomarker that accurately identifies a small and heterogeneous population of non-proliferating premalignant cells in the pancreas, indicating the utility of GLB1 to predict the senescent state in pancreatic preneoplasia [13]. Additionally, increased GLB1 is a valuable marker in formalin-fixed paraffin-embedded tissues for the senescence-like phenotype and associates with improved prostate cancer outcomes [16]. All these evidences support the involvement of GLB1 in carcinogenesis, possibly through regulating cell senescence.

Several limitations may exist in this case-control study. First, because it was a hospital-based study and the cases were from hospitals, there might be inherent selection bias. As a result, it is crucial to confirm these observations in a population-based prospective study. Second, the statistical power for gene-covariate interaction analyses may be limited. Third, since the *P value* for association between the *GLB1* rs4678680 SNP and HCC risk in the current study are not less that 10^−7^, it is possible that these polymorphisms may not be identified by the aforementioned four large scale HCC GWAS in Chinese [7–10].

In summary, our study elucidated that the *GLB1* rs4678680 polymorphism was associated with risk of HBV-related HCC in Chinese populations, highlighting the involvement of *GLB1* and cell senescence in etiology of HCC.

## MATERIALS AND METHODS

### Study subjects

This study consisted of two case-control sets (Supplementary Table S1): (a) Shandong set: 1186 individuals with HBV-related HCC, sex- and age-matched (± 5 years) 508 chronic HBV carriers were recruited at Shandong Cancer Hospital affiliated to Shandong University, Shandong Academy of Medical Sciences (Jinan, Shandong Province, China). (b) Jiangsu set: 620 HBV-related HCC individuals from Huaian No. 2 Hospital (Huaian, Jiangsu Province, China) and sex- and age-matched 1200 chronic HBV carriers as controls. Cases and controls were recruited at Huaian No. 2 Hospital. The case-control sets has been reported previously [17]. A total of 56 pairs of HCC tissue specimens from 56 HCC individuals recruited in this study. All HCC individuals received curative resection in Huaian No. 2 Hospital or Qianfoshan Hospital, Shandong University. Prior to the surgery, no HCC individuals received any local or systemic anticancer treatments. All subjects were ethnic Han Chinese. At recruitment, the written informed consent was obtained from each subject. This study was approved by the institutional Review Boards of Shandong Cancer Hospital, Qianfoshan Hospital and Huaian No. 2 Hospital.

### SNP genotyping

The *GLB1* rs4678680 polymorphism was analyzed by the MassArray system (Sequenom Inc., San Diego, California, USA). *GLB1* rs4678680 PCR primers are 5′-ACGTTGGATGAGTCCAAGCCTGCTTTCTTC-3′ (Forward) and 5′-ACGTTGGATGTCTGCCGAGTTGT TGCAAAG-3′ (Reverse). *GLB1* rs4678680 UEP_SEQ primer is 5′-cctcaTGCTTTCTTCCCTTTTCT-3′. *GLB1* rs4678680 EXT1_SEQ primer is 5′-cctcaTGCTTTCT TCCCTTTTCTG-3′. *GLB1* rs4678680 EXT2_SEQ primer is 5′-cctcaTGCTTTCTTCCCTTTTCTT-3′. A 15% blind, random sample of study subjects was genotyped in duplicates and the reproducibility was 100%.

### Real-time analyses of GLB1 mRNA

SYBR-Green real-time quantity PCR method was used to examine *GLB1* mRNA levels in normal liver tissues as described previously [18–20]. Total cellular RNA was isolated and converted to cDNA using the ReverTra Ace qPCR RT Kit (TOYOBO). Relative gene expression quantitation for *GLB1* and *β-actin* as an internal reference gene was carried out using the ABI 7500 real-time PCR system in triplicates. The primers used for *GLB1* were 5′- GTCTATTCTTCTCCGCTCCT −3′ and 5′- TGTGCTCCATCAGTGGTAA −3′; and for *β-actin* were 5′-GGCGGCACCACCATGTACCCT-3′ and 5′-AGGGGCCGGACTCGTCATACT-3′. The expression of individual *GLB1* mRNA measurements was measured relative to expression of *β-actin* mRNA using the method as described previously [21].

### Statistic analyses

The differences in demographic variables and genotype distributions of the *GLB1* rs4678680 SNP between cases and controls were examined via Pearson's χ^2^ test. The associations between genotypes of *GLB1* rs4678680 and HBV-related HCC risk were estimated by ORs and their 95% CIs computed by logistic regression models. All ORs were adjusted for age, sex, smoking or drinking status, where it was appropriate. Kruskal-Wallis one-way analysis of variance tests were performed to calculate *GLB1* mRNA expression differences between different rs4678680 genotype carriers. A *P* value of less than 0.05 was used as the criterion of statistical significance, and all statistical tests were two-sided. All analyses were performed using SPSS 16.0 (SPSS Inc.).

## SUPPLEMENTARY MATERIALS TABLE


