# Supplementary material for: Association of the GLB1 rs4678680 genetic variant with risk of HBV-related hepatocellular carcinoma
Source: Oncotarget. 2016 Jul 30;7(35):56501–7. doi: 10.18632/oncotarget.10963 (PMC5302931; doi:10.18632/oncotarget.10963)
Supplement: Supplementary file 1 [file oncotarget-07-56501-s001.pdf]

Association of the *GLB1* rs4678680 genetic variant with risk of HBV-related hepatocellular carcinoma

Supplementary Materials

Supplementary Table S1: Distribution of selected characteristics among HBV-related HCC cases and chronic HBV carriers

Variable	Shandong set (Discovery set)			Jiangsu set (Validation set)		
	HCC cases	Chronic HBV carriers	P^a	HCC cases	Chronic HBV carriers	P^a
	No. (%)	No. (%)		No. (%)	No. (%)	
	1186	508		620	1200	
Age (year)			0.626			0.432
≤ 57	627 (52.9)	262 (51.6)		315 (50.8)	633 (52.8)	
> 57	559 (47.1)	246 (48.4)		305 (49.2)	567 (47.2)	
Sex			0.249			0.172
Male	1018 (85.8)	425 (83.7)		531 (85.6)	998 (83.2)	
Female	168 (14.2)	83 (16.3)		89 (14.4)	202 (16.8)	
Smoking			0.104			< 0.001
No	468 (39.5)	222 (43.7)		214 (34.5)	575 (47.9)	
Yes	718 (60.5)	286 (56.3)		406 (65.5)	625 (52.1)	
Drinking			0.133			< 0.001
No	410 (34.6)	195 (38.4)		156 (25.2)	706 (58.8)	
Yes	776 (65.4)	313 (61.6)		464 (74.8)	494 (41.2)	

Note: HBV, hepatitis B virus; HCC, hepatocellular carcinoma.
[a]Two-sided χ^2 test, HCC cases vs. chronic HBV carriers.
